# Development of PBPK model for intra-articular injection in human: methotrexate solution and rheumatoid arthritis case study

**DOI:** 10.1007/s10928-021-09781-w

**Published:** 2021-09-26

**Authors:** Maxime Le Merdy, Jim Mullin, Viera Lukacova

**Affiliations:** Simulations Plus, Inc, 42505 10th Street West, Lancaster, CA 93534 USA

**Keywords:** PBPK, Methotrexate, Product development, Rheumatoid arthritis, Intra-articular

## Abstract

A physiologically based model describing the dissolution, diffusion, and transfer of drug from the intra-articular (IA) space to the plasma, was developed for GastroPlus® v9.8. The model is subdivided into compartments representing the synovial fluid, synovium, and cartilage. The synovium is broken up into two sublayers. The intimal layer acts as a diffusion barrier between the synovial fluid and the subintimal layer. The subintimal layer of the synovium has fenestrated capillaries that allow the free drug to be transported into systemic circulation. The articular cartilage is broken up into 10 diffusion sublayers as it is much thicker than the synovium. The cartilage acts as a depot tissue for the drug to diffuse into from synovial fluid. At later times, the drug will diffuse from the cartilage back into synovial fluid once a portion of the dose enters systemic circulation. In this study, a listing of all relevant details and equations for the model is presented. Methotrexate was chosen as a case study to show the application and utility of the model, based on the availability of intravenous (IV), oral (PO) and IA administration data in patients presenting rheumatoid arthritis (RA) symptoms. Systemic disposition of methotrexate in RA patients was described by compartmental pharmacokinetic (PK) model with PK parameters extracted using the PKPlus™ module in GastroPlus®. The systemic PK parameters were validated by simulating PO administration of methotrexate before being used for simulation of IA administration. For methotrexate, the concentrations of drug in the synovial fluid and plasma were well described after adjustments of physiological parameters to account for RA disease state, and with certain assumptions about binding and diffusion. The results indicate that the model can correctly describe PK profiles resulting from administration in the IA space, however, additional cases studies will be required to evaluate ability of the model to scale between species and/or doses.

**Supplementary Information:**

The online version contains supplementary material available at 10.1007/s10928-021-09781-w.

## Introduction

**Fig. 1:**
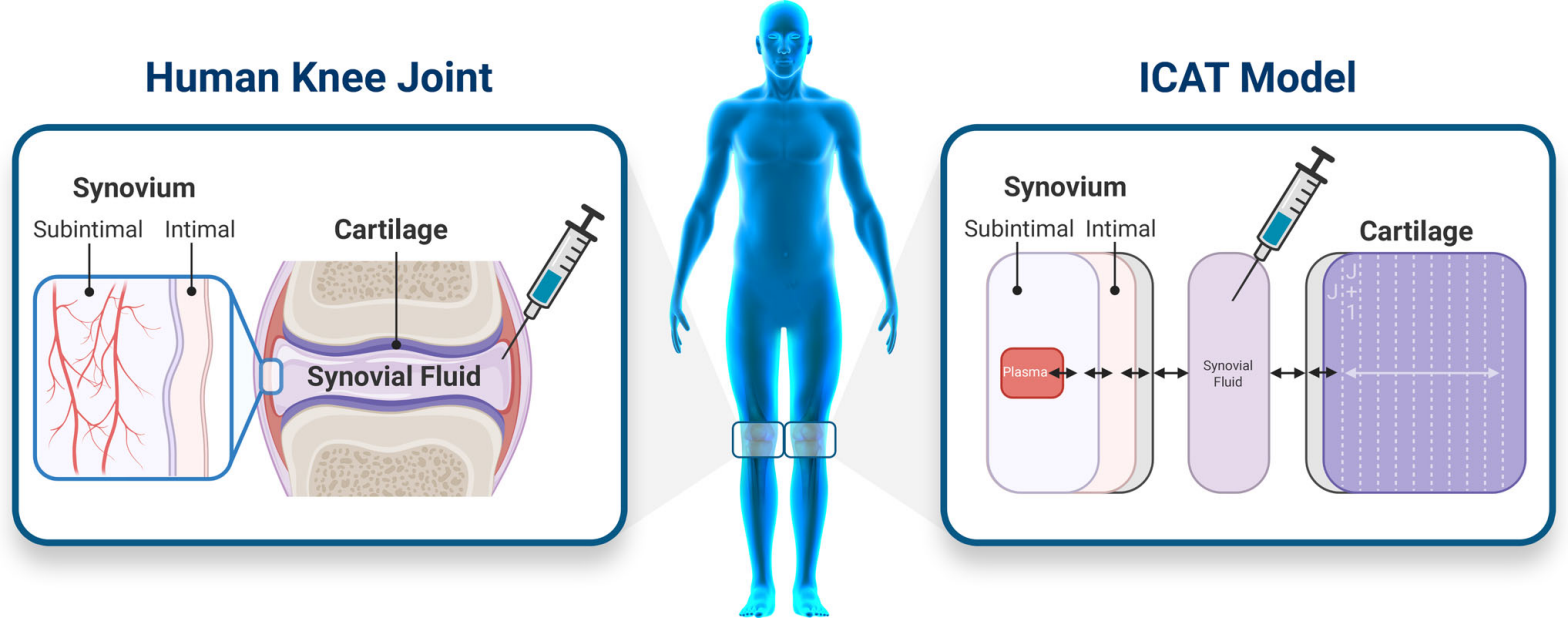
Schematic representation of the human knee joint and the corresponding diagram of the ICAT model. In the ICAT model, synovial fluid, subintimal, intimal and cartilage spaces are identified. The gray compartments represent the boundary layers between synovial fluid and both cartilage and intima compartments

The human knee is one of the largest and most complex joints in the body. It resides at the interface between the femur and the tibia. The fibula and patella are the other bones that complete this joint. The knee joint varies amongst the population but its complex function is constant and consists of the interplay between bony structures, ligaments, tendons, muscles, and joint capsules [1]. To protect the bones’ extremities within the articulation, they are covered with cartilages, an elastic tissue. The inner membrane of the knee joint is the synovium, that is subdivided in the intima and subintimal layers. The intima is in direct contact with the joint cavity, itself filled with synovial fluid, a viscous, non-Newtonian fluid, whose principal role is to reduce the friction between the articular cartilages during movement [2]. The subintimal layers are vascularized with fenestrated capillaries, responsible for the exchange between the systemic circulation and articular tissue [3] (Fig. 1). The knee homeostasis can be altered, resulting in the appearance of clinical symptoms and joint related pathologies such as rheumatoid arthritis (RA) or osteoarthritis. To cure or reduce the symptoms of these pathologies, intra-articular (IA) injections in the synovial fluid that deliver high concentrations of active pharmaceutical ingredients (APIs) to the joint space are routinely utilized [4–6]. Recent IA approaches focus on API that are safe, have significant tissue penetration, and can be administered as controlled or sustained delivery to avoid repeated injections into the joints [7].

RA is a common systemic inflammatory autoimmune disease characterized by painful swollen joints that can severely impair physical function and quality of life. This chronic disease affects between 0.5 and 1% of the United States adult population [8]. Joint swelling in RA reflects synovium inflammation based on immune response activation and is characterized by leucocyte infiltration into the synovial compartment [9]. The intimal lining greatly expands and this tissue lost its fundamental role in joint homeostasis resulting in cartilage and bones damage in advanced RA [10]. The subintima layer volume increases due to the migration and retention of infiltrated cells [11]. Subintima vasculature organization is affected and the distribution of capillary depth (from the synovial surface) increases compared to healthy joints [3]. Synovial fluid volume also increases in RA patients [12]. Methotrexate (MTX) is the first line treatment for patients diagnosed with RA. It is usually administered orally at low doses [8]. Other therapeutic approaches are considered with disease-modifying antirheumatic drugs (DMARDs) or non-steroidal anti-inflammatory drugs (NSAIDs) [5, 6, 8–10, 13]. At the time of this research, 508 active clinical trials were studying RA [14]. These studies focused on new drug therapies and formulations [7], some administered using the IA route of administration (ROA).

Physiologically based pharmacokinetic models (PBPK) were first introduced in the 1970s to support drug product development from preclinical to clinical trials as they can reduce cost and attrition in drug product development [15]. There is great potential for IA PBPK modeling to provide insight into API partitioning in joint tissues that are not accessible and/or are challenging to sample in humans. Because these models are based on physiological parameters, they can predict local and systemic pharmacokinetics (PK) in diseased subjects by accounting for disease progression impact on physiological homeostasis. [16, 17]. Although PK and pharmacodynamic (PD) models have studied APIs effect on RA evolution using clinical or preclinical data [18–20], to our knowledge, no mechanistic absorption and distribution model following IA administration has been developed and published. IA ROA is used for locally acting drug products, and PBPK models have demonstrated their tremendous utility for this type of drug product research, development, and regulatory approval [21–24]. Therefore, a mechanistic PBPK model describing the joint tissue concentration time course following IA administration of an API could be a powerful tool to facilitate development of new therapeutics and improving current practices by better understanding the impact of disease on the API distribution within the joint.

This article describes the application of IA PBPK mechanistic absorption model within GastroPlus® (version 9.8 Simulation Plus Inc., Lancaster, CA, USA) to predict the local and systemic concentrations in the human knee for MTX solution. This study includes (1) development and validation of the systemic PK model following intravenous (IV) and oral (PO) administration of MTX to RA patients (2) validation of the IA model following the single IA administration of MTX to RA patients (3) the investigation of disease state impact on local and systemic MTX concentrations.

## Materials and methods

### Intra-articular model structure

The developed IA model represents the knee articulation as a collection of the following compartments: synovial fluid, cartilage, and synovium, itself subdivided in intimal and subintimal layers. The cartilage compartment is divided into ten sublayers to account for possible concentration gradient due to API diffusion within this tissue. Once the API is present in systemic circulation it can be distributed and cleared form the body. Systemic distribution and clearance can be described either by a PBPK model or a compartmental PK model. A schematic diagram of the developed IA model is shown in Fig. 1.

### Intra-articular model processes and mechanisms

Once the API is dosed in the synovial fluid, the model accounts for dissolution/precipitation in the synovial fluid, diffusion through the synovium, uptake into the systemic circulation from the perfused subintimal layer of the synovium based on the capillary blood flow per unit volume of tissue, and diffusion of the API into the cartilage (Fig. 1). Non-specific binding in all compartments is defined as a fraction unbound to simulate the binding to tissue, protein, or hyaluronic acid. Only the unbound concentration of API in the synovial fluid can diffuse into surrounding tissues (synovium and cartilage).

The model accounts for dissolution/precipitation in the synovial fluid. Unbound concentration in the synovial fluid is the driver for dissolution. For ionizable compounds, both the total amount of material that can dissolve at any point, and the rate of dissolution, partially depends on API aqueous solubility at the synovial fluid pH. Note that once drug has been absorbed, it is assumed to remain in solution. If the concentration of the dissolved drug exceeds the API solubility in the synovial fluid, the API precipitate back to undissolved material.

The boundary condition that determines the amount of API entering the cartilage is based upon the flux equality of unbound API. The unbound fraction of the API at the surface of the cartilage is assumed to be equal to the unbound fraction in the synovial fluid in contact of the cartilage [25]. With this assumption, the loss of API from the synovial fluid to the cartilage can be calculated from Eq. 1. Due to the assumption that the flux is equal on each side of the tissue interface, this equation also represents the flow of API out of the cartilage when $C_{j,u}^{cart}$ > $C_{j,u}^{syn}$.

1 $$\frac{{dM_{syn - cart} }}{dt} = k_{syn,cart} \times SA_{cart} \left( {C_{u}^{syn} - C_{j,u}^{cart} } \right)$$

Here the *M*_*syn-cart*_ is the mass being transferred from synovial fluid to the cartilage, $C_{j,u}^{cart}$ represents unbound API concentration in the cartilage sublayer *j* (cartilage sublayer that is in contact with synovial fluid)*,* $C_{u}^{syn}$ is the unbound API concentration in synovial fluid, k_syn,cart_ is the mass transfer coefficient, and SA_cart_ is the cartilage surface area. The mass transfer coefficient can be estimated by a mass transfer correlation as shown in Eq. 2 [26].

2 $$k_{syn,cart} = \frac{{D_{syn} }}{{L_{cart} }} \times 0.664 \times Re^{\frac{1}{2}} \times Sc^{\frac{1}{3}}$$

Here *D*_*syn*_ is the API diffusivity through the synovial fluid, L_cart_ is the distance between the upper posterior and lower distal cartilage. Based on human measurements of cartilage area and thickness as well as some of the fluid dynamics literature we assumed the synovial gap thickness to be 0.207 × Cartilage Thickness (3,27). Unfortunately, the synovial fluid gap, or gap between the posterior and distal articular cartilage, is not constant, but this falls in the range of expected values. The Reynolds number and Schmidt number are dimensionless transport quantities as shown below (Eq. 3).

3 $$Re = \frac{{\rho vL_{cart} }}{\mu } , \,Sc = \frac{\mu }{{\rho D_{syn} }}$$

Here r is the density, m is the viscosity, and $v$ is the velocity in synovial fluid. The latter is assumed to be 1.5 cm/s based on human computational fluid dynamic calculations backed up by particle image velocimetry measurements [27, 28].

Diffusion through the cartilage sublayers is based on the solution of Fick’s law of diffusion in a planar geometry (Eq. 4).

4 $$\frac{{dC_{j,t}^{cart} }}{dt} = \frac{{D_{cart} }}{{h_{j}^{2} }} \times \left( {C_{j - 1,u}^{cart} - 2 C_{j,u}^{cart} + C_{j + 1,u}^{cart} } \right)$$

In Eq. 4, $C_{j,t}^{cart}$ represents total API concentrations in the cartilage sublayer *j*, respectively; $C_{j - 1,u}^{cart}$ and $C_{j + 1,u}^{cart}$ represent the unbound API concentration in the previous and subsequent sublayer, respectively; *D*_*cart*_ is the API diffusivity through the cartilage; and *h*_*j*_ is the thickness of the cartilage sublayer *j*.

API can also diffuse from the synovial fluid though the synovium. Given the relatively small thickness of the synovium membranes (intimal and subintimal) in relation to the cartilage, these membranes are represented by a single diffusion layer each rather than sublayers like the cartilage.

Mass transfer through the hydrodynamic boundary layer at the interface between the synovial fluid and intima is calculated based on Eq. 5. The mass transfer coefficient, *k*_*syn,int*_ accounts for both diffusion through the synovial fluid boundary layer and the intimal tissue [27]. For a healthy joint, the intimal layer is a non-vascular 18 micron thick cellular membrane directly in contact with the synovial fluid. This serves as the final diffusive barrier before API reaches the larger 282 micron sub-intimal layer where it can enter systemic circulation through fenestrated capillaries. Again, only unbound API is assumed to diffuse through these tissues. Equations 5 and 6 describe the diffusion through the intimal and subintimal layers.

5 $$\frac{{dM_{syn - int} }}{dt} = k_{syn,int} \times SA_{int} \left( {C_{u}^{syn} - C_{u}^{int} } \right),\, where\; k_{syn,int} = \frac{1}{{\frac{1}{{k_{syn,bl} }} + \frac{{h_{int} }}{{D_{int} }}}}$$

6 $$\frac{{dM_{int - subint} }}{dt} = \frac{{D_{subint} }}{{h_{subint} }} \times SA_{subint} \left( {C_{u}^{int} - C_{u}^{subint} } \right)$$

Here *M*_*syn-int*_ and *M*_*int-subint*_ is the mass transfer from synovial fluid to intimal membrane and intimal membrane to subintimal membrane, respectively, *D*_*int*_ and *D*_*subint*_ are the diffusion coefficients in the intimal and subintimal membrane, respectively, h_int_ and h_subint_ are the thickness of the intimal and subintimal membranes, respectively, k_syn,int_ is the mass transfer coefficient in the synovium, k_syn,bl_ is the mass transfer coefficient for the synovial fluid to membrane hydrodynamic boundary layer, *SA*_*int*_ and *SA*_*subint*_ are the equal surface areas of intimal and subintimal membrane, respectively, and $C_{u}^{int}$ and $C_{u}^{subint}$ are the unbound concentrations in intimal and subintimal layer, respectively. These equations are bi-directional in that if the concentration gradients change, API can flow in either direction.

The mass transfer coefficient in the synovial fluid boundary layer (k_syn,bl_) is estimated using the Reynolds Number and Schmidt number as shown in Eq. 7, which is similar to Eq. 2. However, in this case, the length scale is estimated by assuming the synovium is a cylinder with the characteristic height of that cylinder (L_syn_). The mass transfer coefficient for the hydrodynamic boundary layer between the synovial fluid and membrane can then be calculated (Eq. 7).

7 $$k_{syn,bl} = \frac{{D_{syn} }}{{L_{syn} }} \times 0.664 \times Re^{\frac{1}{2}} \times Sc^{\frac{1}{3}}$$

Here *D*_*syn*_ is the API diffusivity through the synovial fluid. The Reynolds number and Schmidt number are dimensionless transport quantities as shown in Eq. 3. Diffusion coefficients in different tissues can be estimated by multiple equations either based on compound or physiologic properties. For all tissues, these coefficients can be set equal to the API diffusivity in water. Optionally, diffusion coefficient can be estimated by Eq. 8 that relates lipophilicity or logD at pH 7.4. This relationship was developed for oral cavity tissues [24], and can be utilized here as an approximation due to lack of measured diffusion coefficients in intra-articular tissues.

8 $$\;D_{i} = \left\{ {\begin{array}{*{20}l} {10^{{ - 0.0803 \times LogD\left( {7.4} \right)^{2} + 0.5006 \times LogD\left( {7.4} \right) - 6.7316,}} } & {LogD\left( {7.4} \right) \le 3} \\ {10^{ - 5.9514,} } & {LogD\left( {7.4} \right) > 3} \\ \end{array} } \right.$$

The synovial fluid is a high viscosity fluid, which presumably, would slow down the diffusion rate. For this fluid, Stokes–Einstein equation [26], which relates diffusivity to solute radius and solvent viscosity, can be applied (Eq. 9).

9 $$D_{syn} = \frac{{k_{B} T}}{6\pi \eta r}$$

Here *D*_*syn*_ is the diffusion coefficient of API in synovial fluid, *k*_*b*_ is Boltzmann’s constant, *T* is temperature (in kelvin), *η* is the dynamic viscosity of media, and *r* is the API molecular radius.

The unbound concentration in the various joint tissues is calculated from fraction unbound according to the general Eq. 10. Each tissue has its own fraction unbound since different nonspecific binding may be applicable in each tissue.

10 $$C_{u} = f_{ut} \times C_{T}$$

Here *C*_*u*_ and *C*_*T*_ are the unbound and total concentration, respectively, in any tissue and f_ut_ is the fraction unbound.

The API uptake into systemic circulation is an instant partitioning between the unbound concentration in plasma and the unbound concentration in the subintimal layer of the synovium and its rate is dependent on the blood flow rate through the fenestrated capillaries of the synovial subintimal membrane as described by Eq. 11.

11 $$SystRate \, = Q \times R_{bp} \times \left( {C_{u}^{subint} \times \frac{1}{{f_{up} }} - C_{p} } \right)$$

Here Q is the blood flow rate through the subintimal layer, Rbp is API blood/plasma concentration ratio, fup is fraction of API unbound in plasma, $C_{u}^{subint}$ is unbound API concentration in subintimal membrane, and Cp is total API concentration in plasma.

All equations’ parameters nomenclature and units are summarized in supplementary material.

### Intra-articular model physiological parameters

The physiological parameters for healthy and RA human knee joint were collected from literature and are summarized in Table 1.

**Table 1:** IA physiological parameters describing a healthy or RA joints

Parameter	Value	Reference
IA healthy physiology		
Synovial fluid volume	2.21 mL	[30, 31]
Synovial fluid pH	7.7	[29]
Synovial fluid viscosity	389.5 cP	[2, 29]
Synovium area	277 cm^2^	[3]
Cartilage area	148 cm^2^	[3]
Cartilage thickness	2.5 mm	[3]
Intima layer thickness	18 µm	[3, 32–35]
Subintima layer thickness	282 µm	[3, 32]
Blood flow rate	0.63 mL/min/mL synovium	[3]
IA RA physiology		
Synovial fluid volume	30 mL	[12]
Intima layer thickness	162 µm	[33, 36]
Subintima layer thickness	705 µm	[3]

Synovial viscosity is significantly reduced with age and disease state [2, 29]. Synovial fluid mean pH is around 7.7 and does not vary with age [29]. The subintimal layer of the synovium is the only vascularized tissue of the joint. The microvascular architecture of the joint has been extensively described in literature and the blood flow in the model was extracted from Levick [3].

Although literature information was accessible for synovial fluid volume [30, 31], synovium, cartilage surface area, and cartilage thickness in healthy conditions [3], the synovium thickness parameters had to be extrapolated from multiple sources. The intima layer is composed of 1–2 layer(s) of fibroblasts [3, 32–35]. Because a human fibroblast thickness is considered to be 10 to 15 µm, an average intima thickness of 18 µm was defined for the healthy knee physiology parameter. This value was confirmed by Levick et al. who measured the distribution of capillary depths in a normal human knee and showed that less than 4% of the capillaries are present within the first 20 microns of the synovium [3]. Because the intima is avascular, this measurement confirms the value chosen for intima thickness. Levick et al. determined all capillaries are distributed within 300 microns from the synovium surface. Because the capillaries are only present in the subintima, a value of 282 microns for the healthy human knee physiology was assumed for its thickness.

Parameters for the RA physiology had to be extrapolated from multiple sources. Synovial fluid volume measured in five RA patients is greatly increased from the baseline value and ranges between 15 and 60 mL [12]. Therefore, an average value of 30 mL was selected for model simulations. John Hopkins Arthritis Center indicates that during RA, intima thickness is increased as it is now composed of 8–10 cells layers [33] representing an 8 to tenfold increase. Multiple publications report a similar increase in intima thickness [8, 9, 34]. Therefore, a scaling factor of 9 for the RA physiology was applied to the intima thickness parameter. Subintima layer is infiltrated by multiple immune cells, including T and B lymphocytes, macrophages, mast cells, and mononuclear cells that differentiate into multinucleated osteoclasts as RA progresses. Its thickness is increased, however not in similar proportion as the intima. Levick et al. measured a threefold reduction in the capillary density in the subintima for a RA joint compared to healthy joint [3] which might indicate the volume of this tissue is increased by a similar ratio. However, new blood capillaries are created during the disease progression and such a ratio cannot be used directly to estimate the subintimal thickness. A conservative approach was used and a value of 705 microns (2.5 fold) was selected for model simulations of RA physiology. After parameter scaling, the synovium thickness is 869 microns, representing a 2.9 fold increase, aligned with published value for other human joints affected by RA [36].

### MTX IA solution case study

GastroPlus® (version 9.8 Simulation Plus Inc., Lancaster, CA, USA) was used for computer simulation of MTX biodistribution after intraarticular injection in human knee. Model structure integrates a three-compartment PK model for systemic distribution and clearance, an IA Compartmental Absorption & Transit (ICAT™) model describing IA disposition and transfer into the systemic circulation, and an Advanced Compartmental Absorption & Transit (ACAT™) model to describe the oral absorption of MTX in RA patients. Input parameters for the compound are listed in Table 2.

**Table 2:** Summary of MTX parameters implemented in the model

Parameter	Value	Reference
MTX physicochemical properties		
logP	− 1.85	[37]
Aqueous diffusion coefficient	0.62 × 10^–5^ cm^2^/s	ADMET predictor^*a*^
Reference solubility	0.16 mg/mL @ pH = 4.2	ADMET predictor^*a*^
Human effective permeability (P_eff_)	0.65 × 10^–4^ cm/s	Fitted
Particle radius	25 μm	GastroPlus default
Precipitate radius	1 μm	GastroPlus default
Drug particle density	1.2 g/mL	GastroPlus default
Mean precipitation time	900 s	GastroPlus default
Blood:plasma concentration ratio (R_bp_)	0.79	ADMET predictor^*a*^
Plasma protein binding (Fup)	66%	[38]
Adjusted fup	66%	GastroPlus algorithm^*b*^
MTX distribution and elimination		
Renal clearance	84.9 mL/h/kg	Fitted
Central volume	64.34 mL/kg	Fitted
k12	2.74 1/h	Fitted
k21	0.895 1/h	Fitted
k13	0.196 1/h	Fitted
k31	0.035 1/h	Fitted
MTX IA		
Free fraction in joint tissues	66%	Assumed
Diffusivity in joint tissues	0.62 × 10^–5^ cm^2^/s	Assumed

^a^Predicted using ADMET Predictor v9.5

^b^Adjusted Fup was calculated from experimental Fup and logD @ pH = 7.4 using the default GastroPlus equation

In simulating PK from clinical studies, the demographics of the study population (body weight) of each study was matched as closely as possible, dependent upon available information within the publications. The dosing regimen (dose, ROA, administration schedule) were also set to match publish information. Published MTX plasma concentration following the IV and PO administration of different doses in RA patients [38–42] were used to fit compartmental PK parameters using the PKPlus™ module and the human effective permeability (Peff). Compartmental PK model was fitted against plasma concentration time profile after 15 mg IV administration [39–41]. The fitted parameters were validated using external datasets after 10 mg [42] and 10 mg/m^2^ [38] IV administration of MTX. Peff was fitted against plasma concentration time profile following PO administration of 15 mg dose [39–41]. The fitted parameters were validated using an external dataset after 10 mg PO administration of MTX [38]. If the model could describe the observed data of the external validation dataset, then the model was considered validated. Elimination was assigned to renal clearance based on the MTX physiological clearance mechanism [43]. The ICAT model was then used to simulate the synovial fluid and plasma concentration time courses following single IA administration of 5 mg of MTX solution [44]. The reported MTX blood concentration has been transformed to plasma concentration using the MTX blood to plasma concentration ratio. The injection volume was not described in the publication, therefore two milliliters, the typical volume for IA administration for products currently in the U.S. market [45], was assumed.

Parameter sensitivity analysis (PSA) was performed to better understand the impact of physiological changes on MTX plasma and synovial fluid exposure. The model was then used to estimate local and systemic MTX PK for RA patients at different stages of disease progression.

## Results

**Fig. 2:**
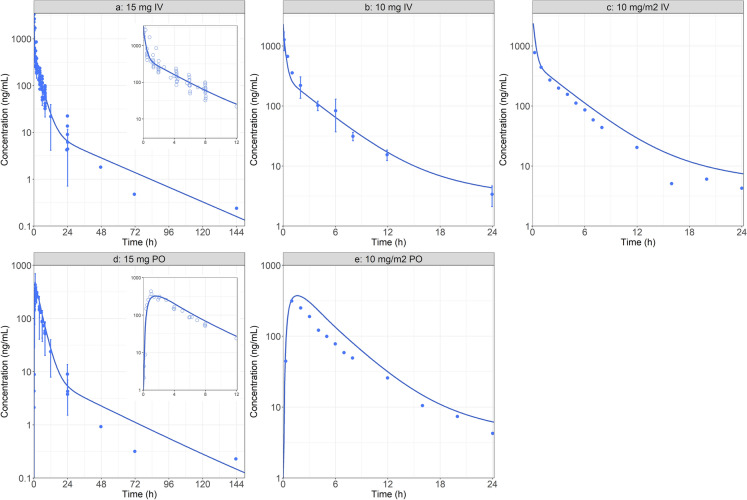
Model simulations following the administration MTX in RA patients: **a** Model development: 15 mg IV bolus [39–41]. **b** External validation 1: 10 mg IV bolus [42]. **c** External Validation 2: 10 mg/m2 IV bolus [38]. **d** Oral absorption model development: 15 mg PO [39–41]. **e** External validation 3: 10 mg/m2 PO [38]. For figures **a** and **d**, a zoom on the initial 12 h is provided as an insert

The baseline distribution and elimination model for MTX was derived from plasma concentration data following 15 mg IV bolus administration from three different studies with RA patients presenting fairly similar population metrics (age, body weight (BW)) [39–41]. The observed concentrations were best described by a three-compartment PK model (Fig. 2A). MTX plasma concentrations following the IV administration of fixed 10 mg dose to RA patients were used as the first external model validation [42]. The predicted plasma concentration time profile matched the observed data very well (Fig. 2B). A second external validation was performed by simulating the plasma concentration time course following the IV administration of 10 mg/m2 to RA patients, assuming an average 1.73 m^2^ of surface area [38]. Once again, the model predicted the observed data reasonably well (Fig. 2C). Based on the model fit and two external validations, the systemic distribution and elimination three-compartment model, specific to RA patients, was deemed acceptable and the following simulations were performed.

The ACAT and compartmental PK model were subsequently used to describe the MTX plasma concentration following the PO administration of 15 mg MTX in RA patients. The observed data were pooled from three different studies [39–41]. In the absence of an in vitro permeability value for MTX, initial simulations utilized in silico predicted value based on MTX 2D structure. This resulted in significant underprediction of the observed data, likely due to effect of folate transporters on MTX absorption in vivo [46]. Human Peff was fit to describe the data accordingly (Fig. 2D). For this model, the fitted Peff represents both passive and active absorption by folate transporters. As an external validation, the model was used to simulate MTX the plasma concentration time course following the PO administration of 10 mg/m2 to RA patients [38]. This simulation overlaid nicely with the observed data and therefore the oral absorption model was accepted (Fig. 2E). Table 3 presents the PK metrics ratios demonstrating the goodness of fit of the baseline model for IV and PO administration.

**Table 3:** Observed and simulated (both baseline and RA physiologies) PK metrics in plasma

Study	Dose	Route	AUC (ng.h/mL)			C_max_ (ng/mL)		
			Obs	Pred	Ratio	Obs	Pred	Ratio
Model development	15 mg	IV	3351.3	2613.4	0.78	NA	3479.6	NA
External validation 1	10 mg	IV	1932.8	1690.6	0.87	NA	2319.8	NA
External validation 2	10 mg/m2	IV	2002.8	2793.3	1.39	NA	4013.2	NA
Oral absorption model development	15 mg	PO	1840.6	1951.8	1.06	367.38	323.25	0.88
External validation 3	10 mg/m2	PO	1398	2071.9	1.48	313.68	372.87	1.19

**Fig. 3:**
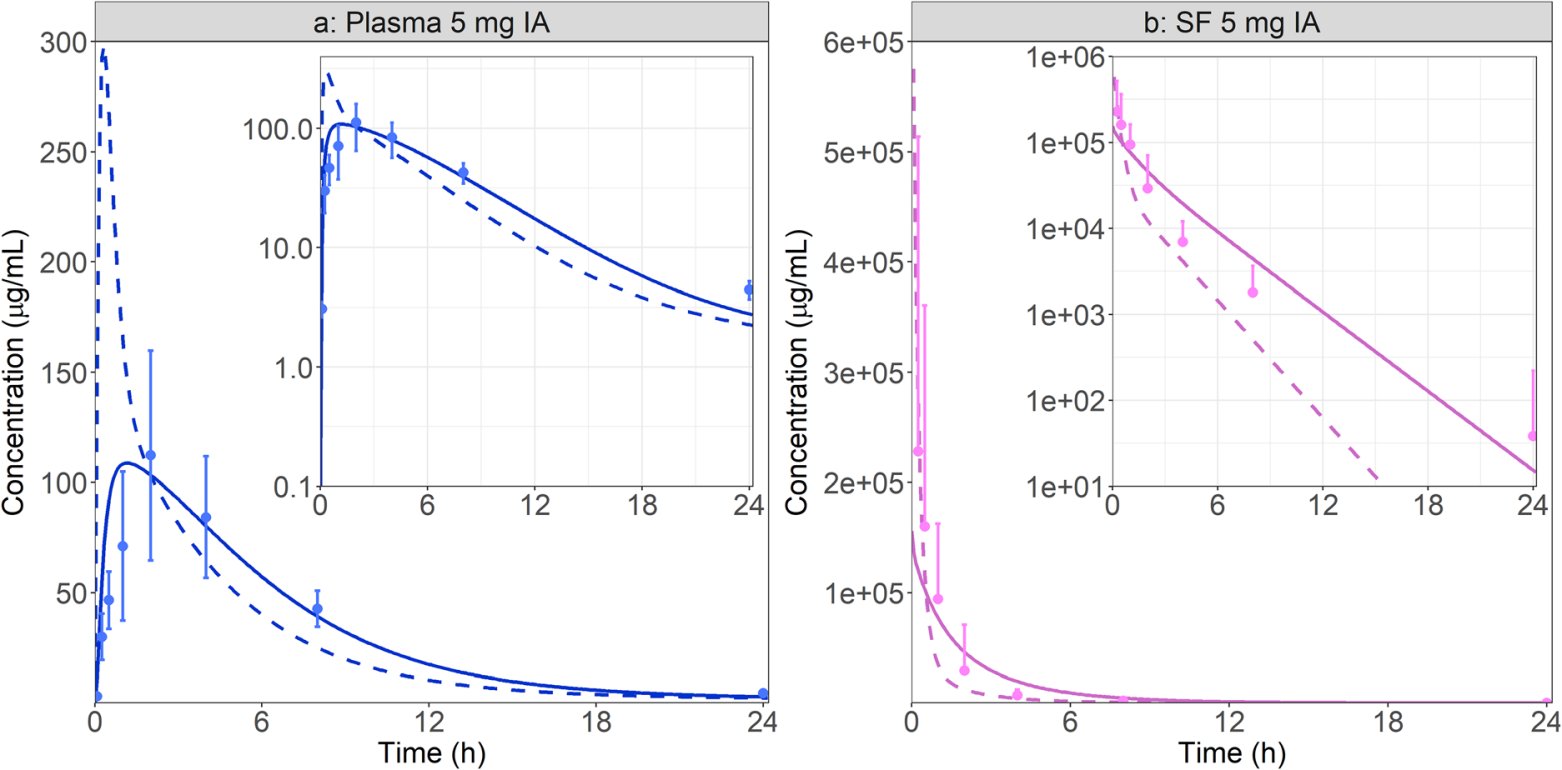
Plasma (**a**) and synovial fluid (**b**) concentration time course following the administration of 5 mg MTX in the synovial fluid of a human knee [44]. Dashed lines represent the baseline simulation with the default healthy joint physiology and solid lines represent the simulation with the specific RA physiology. Both linear and log scales are presented. For SF observed data, only the above standard deviations are presented to enhance results legibility

To evaluate the effect of knee joint physiology linked with RA, the simulations were done with both healthy and RA physiology. Synovial fluid and blood data following the IA administration of 5 mg of MTX solution in RA patients were extracted from literature [44]. Plasma concentrations were calculated using the digitized blood concentrations and MTX blood to plasma ratio (Table 2). The initial model used the default physiology of a healthy knee joint in human. Free fraction in all joint tissues was assumed similar to plasma free fraction. MTX diffusivity in synovial fluid, intima, subintima, and cartilage was set to MTX diffusivity in water (0.62 × 10^5^ cm^2^/s). Despite describing well, the initial synovial fluid concentrations, the model greatly overestimated the initial rate of MTX transfer from the joint cavity to the plasma compartment, as it can be observed in Fig. 3. Although the plasma and synovial fluid AUC_0-25_ are well predicted, plasma Cmax is overestimated nearly threefold and Tmax is underestimated more than sixfold, (Table 4).

**Table 4:** Observed and simulated (both baseline and RA physiologies) PK metrics in plasma and synovial fluid following IA MTX administration [44]

	Plasma			Synovial fluid	
	Cmax (ng/mL)	AUC_0-25_ (ng.h/mL)	Tmax (h)	Cmax^a^ (µg/mL)	AUC_0-25_ (µg.h/mL)
Observed^b^	108.3	960.6	2.0	228.0	270.8
Baseline simulation	298.3	801.2	0.3	272.7	260.5
Ratio_Healthy_	2.75	0.83	0.17	1.20	0.96
RA simulation	108.8	796.5	1.2	120.2	279.6
Ratio_RA_	1.00	0.83	0.58	0.53	1.03

^a^For synovial fluid, the simulated Cmax represents simulated concentration at the time of the first measurement to allow comparison with the observed data

^b^Observed PK metrics or obtained in RA patients

RA is known to significantly alter the joint physiology by increasing the synovial fluid volume and synovium thickness. An RA physiology was created by setting the synovial fluid volume to 30 mL [12] and modifying the intima and subintima layer thickness to 162 and 705 µm [3, 10, 11]. All other API and physiological parameters were kept at the default values for healthy knee joint due to the lack of clear public information of their changes during RA progression. Inclusion of major RA physiological consequences in the PBPK model greatly improved the model predictions (Fig. 3). Similar to the baseline simulation, both plasma and synovial fluid AUCs are well predicted (within 1.25-fold of the observed AUCs_0-25_). However, the plasma Cmax and Tmax are better described with the new diseased state considering the RA pathophysiology (Table 4). Nevertheless, synovial fluid (SF) Cmax prediction seems to be impaired by those changes, even if the predicted concentration values remain within the observed standard deviation. The shapes of the plasma and SF concentration time courses are also better described with the RA physiology.

**Fig. 4:**
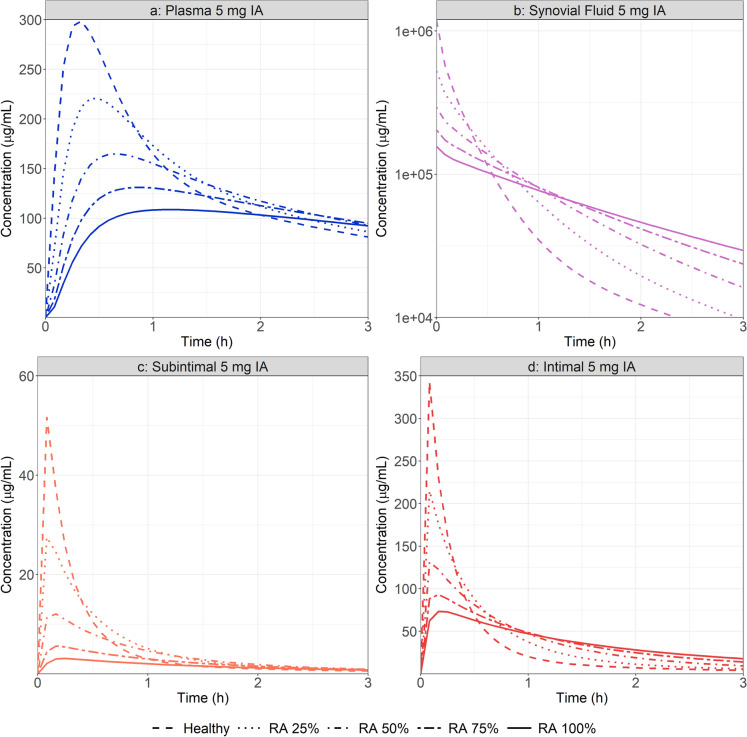
Simulated plasma (**a**), synovial fluid (**b**), subintimal (**c**) and intimal (**d**) concentration time course following the administration of 5 mg MTX in the joint cavity at different stages of RA progression

The validated PBPK model for MTX IA administration was first used to assess the effect of physiological changes during RA disease progression on both local and systemic MTX PK. Plasma, intima, subintima, and synovial fluid concentration time courses were simulated for a healthy joint and at 25, 50, 75 and 100% of RA progression (Fig. 4). To simulate different degrees of RA progression, the three physiological parameters (synovial fluid volume, intima and subintima layers thickness) were scaled mostly proportionally (with the exception of subintima thickness in early RA), with 100% representing the final RA values as listed in Table 1.

The absolute values of parameters for different RA degrees are summarized in supplementary material. As expected, RA progression greatly impacts the transfer of MTX from the site of administration to plasma, based on the reduction of plasma Cmax and extended Tmax. Also, due to increased intima, subintima, and synovial fluid volume, MTX Cmax is significantly lowered in the synovium sublayers. Because this is the site of action for this API [46], RA stage could potentially influence its pharmacodynamic response. RA disease progression may then require dose adjustment in order to achieve the expected MTX effect.

**Fig. 5:**
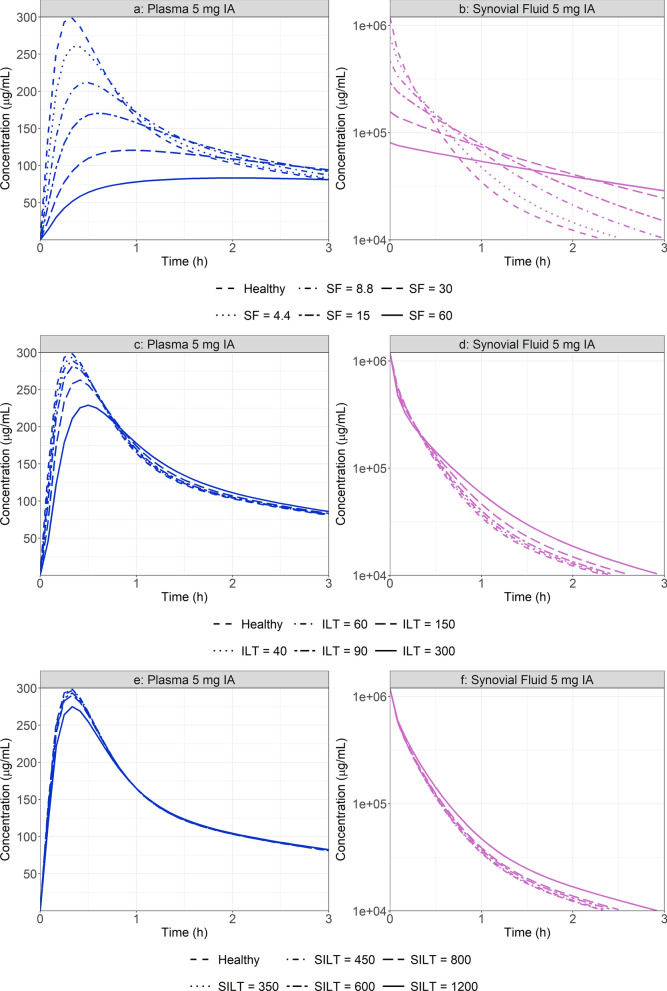
Synovial fluid (SF) volume (**a**, **b**), intima layer thickness (ILT) (**c**, **d**) and subintima layer thickness (SILT) (**e**, **f**) PSA on plasma and synovial fluid concentration time course

To identify the main physiological driver of RA on MTX local and systemic concentration time courses, PSA investigating the impact of changes in individual knee joint physiological parameters was performed. Values for synovial fluid volume, intima, and subintima layer thickness were adjusted. In each case, a baseline simulation was performed using the healthy joint parameter values (Table 2). A range of parameters starting at the baseline physiological values extending beyond the ones used in the specific RA physiology (Table 2) were tested, and the tested values are presented in supplementary material. The synovial fluid volume showed the most significant effect on both MTX local and systemic concentration (Fig. 5). As volume increases, initial synovial concentration decreases due to the dilution of the administered dose. This impacts the initial concentration gradient between the site of administration and surrounding tissues as well as the transfer of API between the synovial cavity and the plasma. With increasing synovial fluid volume, plasma Cmax is reduced while Tmax is extended with a fairly constant AUC_0-25_. Despite small changes on plasma Cmax, PSA demonstrated that any single change of intima or subintima layer thickness has a minimal impact on both local and systemic exposure.

## Discussion

Understanding of the knee joint physiology and its impact on API PK after intraarticular injection is critical for development of new APIs aiming to cure RA and other specific diseases affecting human joints. This research proposes the use of modeling and simulation to support drug product development for API administered intra-articularly. PBPK models for locally acting drug products are actively promoted by regulatory authorities as a necessary tool to address the local and systemic safety and efficacy concerns for both new and generic drug products [23, 47, 48].

The proposed model structure is a simplified representation of the joint, by considering the synovial fluid, cartilage, and synovium. A model is not meant to exactly represent the described biological system. Indeed, modeling is the process of interpreting multifactorial data including disease state, disease progression, API characteristics, and their interplay in the form of a mathematical algorithm [49]. Within the proposed structure, the synovium is divided into two distinct layers, the intima and the vascularized subintima. Within the subintima, the API can diffuse into the systemic circulation and then be distributed in the rest of the body to be eliminated. The model organization does not include some anatomical structures of the knee joint such as the meniscus and bones: patella, femur, tibia, and fibula [1]. Also, this model considers the physiological parameters constant across the entire joint cavity, whereas is it known, the synovium thickness varies based on specific localization within the knee joint, e.g., retropatellar, suprapatellar, infrapatellar areas [32]. Because of the limited human PK data available for drug product administered directly in the knee cavity, creating a more complex model structure would make that model significantly harder to validate. Therefore, the proposed structure is an appropriate initial step to describe the API transfer from the knee cavity into the systemic circulation as well as predicting the concentration in the synovium sublayers.

Each tissue that is represented in the model structure (Fig. 1) is defined by physiological parameters such as volume (for the synovial fluid), the surface area and thickness (for the synovium and cartilage). Synovium layer thickness parameters had to be extrapolated from multiple sources as direct measurements could not be identified in literature. MRI measurements of synovium thickness in 14 year old children demonstrated the thickness varies from 0 to 1.8 mm, based on the joint location, with an average of ~ 400 microns, which aligned with the value used in our model for the total synovium. However, this MRI study also demonstrates the heterogeneity in synovium thickness based on joint location. Therefore, local differences in API exchanges between the synovial fluid and the synovium are to be expected. In future work, ICAT model structure could be modified to account for those regional differences, if case studies in healthy subjects are available for its validation. All other physiological parameters used in the healthy knee physiology, e.g., pH and viscosity, had multiple concurrent sources and could be used as such in the model.

To validate the new ICAT model, MTX was chosen as a case study as this API had sufficient in vitro and in vivo data. MTX was administered using the IA route of administration to three patients (total of seven injections) affected by RA [44]. This inflammatory autoimmune disease is known to induce swollen joints, although the degree of disruption varies significantly between subjects. To capture the RA effect, the synovial fluid volume and both intima and subintima thickness were modified as they represent the most common and significant changes across patients. This model with parameter values representing RA physiology was able to predict both MTX local and systemic observed PK profiles. PSA on individual parameters demonstrated that the changes in intima or subintimal layer thickness alone has a minimal impact on API transfer between the synovial fluid and the synovium, therefore, mitigating the risk of their parameter estimation. Despite good model predictions of observed MTX data, it is important to point out that some RA physiological changes such as cartilage and bone destruction [9] were not implemented, due to the lack of published information, in the model and could potentially be important for predictions of other compounds. Cartilage damages are often present during RA evolution, but the damages progression could not be identified yet. Protein amounts in the synovial fluid and other joint tissues could also potentially be affected by the RA mediated inflammatory reaction, but this was not considered in the MTX case study. However, the current model, even with those limitations, already provides significant insight into the PK changes due to disease progression. More case scenarios including more significant damage to cartilage thickness or changes in protein binding will be tested in the future by the model and validated against observed data for late RA stages when additional studies are published.

Several options for estimation of diffusion coefficient of the joint tissues were implemented and tested: (1) The Stokes–Einstein model that accounts for effect of solvent viscosity on solute diffusion coefficient [26] (2) a simple quantitative structure property relationship (QSPR) for tissue diffusion coefficient estimate from LogD at pH 7.4 [24] (3) assume the compound diffusion coefficient is the same as diffusion coefficient in water. Synovial fluid viscosity is 389.5 cP [2, 29], which is almost 3 orders of magnitude higher than water viscosity of 0.69 cP at 37 °C. This results in predicted diffusion coefficient in synovial fluid almost 3 orders of magnitude lower than that in water based on Stokes–Einstein theory. However, that estimate is not consistent with measurements of diffusion in both cartilage and synovial fluid reported in several publications for multiple molecules [50–52], which indicate that the diffusivity would be more similar to that in water than the one estimated from Stokes–Einstein equation. The theory behind this is that the synovial fluid is not homogenous medium but rather the hyaluronic acid polymer chains create a hydrogel type network of polymer chains. If the polymer network is diluted, the water and solutes can freely diffuse between polymer molecules in the aqueous phase which has a viscosity similar to native water at the microscopic level. The polymer network provides the bulk viscous properties but does not hinder diffusion of small solutes. Therefore, MTX diffusivity in all joint tissues was considered similar to that in water. Protein binding is also a critical factor for the transfer of the API between tissues. MTX fraction unbound in joint tissues was assumed equal to plasma free fraction. For synovial fluid, this assumption is supported by albumin concentration similar to that in plasma [31]. For other joint tissues, albumin concentration information could not be identified, and this assumption will need to be validated in simulations of other compounds and/or adjusted if more physiological information becomes available in the future.

To conclude, a PBPK model utilizing a simplified human joint structure was developed. All physiological parameters were directly extracted or derived from information available in literature. The model was validated using the MTX case study. It was shown that by incorporating the RA disease state on the most sensitive physiological parameters of the knee joint, the local and systemic MTX concentrations could be simulated well without any other model adjustments. Model predictions will be enhanced in the future by considering other consequences of RA on joint physiologies, such as cartilage damage or protein concentrations modification in the joint tissues. In addition, this model for solution dose of MTX to synovial fluid will be extended in the future to include more cases studies and extended validation that will support the investigation of joint heterogenicity, intersubject variability, interspecies scaling, and predictions for other human joints.

## Supplementary Information

Below is the link to the electronic supplementary material.

Supplementary file1 (DOCX 22 kb)
