# Supplementary material for: Development of PBPK model for intra-articular injection in human: methotrexate solution and rheumatoid arthritis case study
Source: J Pharmacokinet Pharmacodyn. 2021 Sep 26;48(6):909–22. doi: 10.1007/s10928-021-09781-w (PMC8604827; doi:10.1007/s10928-021-09781-w)
Supplement: Supplementary file 1 — Supplementary file1 (DOCX 22 kb) [file 10928_2021_9781_MOESM1_ESM.docx]

Development of PBPK Model for Intra-articular Injection in Human: Methotrexate Solution and Rheumatoid Arthritis Case Study.

Maxime Le Merdy*^1^, Jim Mullin^1^, Viera Lukacova^1^

*1: Simulations Plus, Inc., 42505 10th Street West, Lancaster, California 93534, USA.*

*Maxime Le Merdy and Jim Mullin contributed equally to the realization of this research project.*

*Corresponding author: Maxime Le Merdy

*Simulations Plus, Inc., 42505 10th Street West, Lancaster, California 93534, USA.*

Phone: +1-661-723-7723, Email: [maxime@simulations-plus.com](mailto:maxime@simulations-plus.com)

**Running title:** Intra-articular PBPK modeling of methotrexate solution.

**Keywords:** PBPK, methotrexate, product development, rheumatoid arthritis, intra-articular

Table 5: Nomenclature and units in order of appearance.

|  | Description | Units |
| --- | --- | --- |
| $\frac{dM_{syn-cart}}{dt}$ | Rate of mass transfer from the synovial fluid to the cartilage | mg/s |
| $k_{syn,cart}$ | Mass transfer coefficient from synovial fluid to cartilage | cm/s |
| $SA_{cart}$ | Surface area between cartilage and synovial fluid | cm^2^ |
| $C_{u}^{syn}$ | Unbound concentration of drug in synovial fluid | mg/mL |
| $C_{j,u}^{cart}$ | Unbound concentration in cartilage at node *j* | mg/mL |
| $D_{syn}$ | Diffusivity in the synovial fluid | cm^2^/s |
| $L_{cart}$ | Distance between the posterior and anterior cartilage | cm |
| $Re$ | Reynolds number | dimensionless |
| $Sc$ | Schmidt number | dimensionless |
| $\rho$ | Density | g/mL |
| $v$ | Velocity | cm/s |
| $\mu$ | Viscosity | poise |
| $h_{j}$ | Thickness of cartilage node *j* | cm |
| $\frac{dM_{syn-int}}{dt}$ | Rate of mass transfer from synovial fluid to intimal membrane | mg/s |
| $k_{syn,int}$ | Mass transfer coefficient from synovial fluid to intimal membrane | cm/s |
| $SA_{int}$ | Surface area of intimal membrane | cm^2^ |
| $C_{u}^{syn}$ | Unbound drug concentration in synovial fluid | mg/mL |
| $C_{u}^{int}$ | Unbound drug concentration in intimal membrane | mg/mL |
| $k_{syn,bl}$ | Mass transfer coefficient in unstirred water layer of synovial fluid | cm/s |
| $h_{int}$ | Thickness of intimal membrane | cm |
| $D_{int}$ | Diffusivity of drug in intimal tissue | cm^2^/s |
| $\frac{dM_{int-subint}}{dt}$ | Rate of mass transfer between intimal and sub-intimal membrane | mg/s |
| $D_{subint}$ | Diffusivity in the subintimal membrane tissue | cm^2^/s |
| $h_{subint}$ | Thickness of subintimal membrane | cm |
| $SA_{subint}$ | Surface area of subintimal membrane | cm^2^ |
| $C_{u}^{subint}$ | Unbound concentration in the subintimal membrane | mg/mL |
| $L_{syn}$ | Length scale of unstirred water layer in synovial fluid | cm |
| $k_{B}$ | Boltzmann’s constant | J/K |
| $T$ | Temperature | K |
| $\eta$ | Kinematic viscosity | centistoke |
| $r$ | Molecular radius | cm |
| $C_{u}$ | Unbound concentration | mg/mL |
| $f_{ut}$ | Fraction unbound in tissue | unitless |
| $C_{T}$ | Total concentration in tissue | mg/mL |
| SystRate | Systemic absorption rate | mg/s |
| $Q$ | Tissue blood flowrate | mL/s |
| $R_{B:P}$ | Blood to plasma concentration ratio | unitless |
| $Fup$ | Fraction unbound in plasma | unitless |
| $C_{P}$ | Concentration of drug in plasma | mg/mL |

Table 6: Synovial fluid volume, intima and subintima layer thickness parameters values used for parameter sensitivity analysis investigating the consequences of physiological changes during rheumatoid arthritis disease progression on both methotrexate local and systemic pharmacokinetic.

|  | Synovial fluid volume (mL) | Intima (µm) | Subintima (µm) |
| --- | --- | --- | --- |
| *healthy* | 2.2 | 18 | 282 |
| *25%* | 7.5 | 40.5 | 282* |
| *50%* | 15 | 81 | 352.5 |
| *75%* | 22.5 | 121.5 | 528.75 |
| *100%* | 30 | 162 | 705 |
| *minimum value of 282. Initial stages of rheumatoid arthritis only affect the intima layer. | | | |

Table 7: Synovial fluid volume, intima and subintima layer thickness parameters values used for parameter sensitivity analysis investigating the impact of knee joint physiological changes on methotrexate local and systemic exposure.

|  | Synovial fluid volume (mL) | Intima (µm) | Subintima (µm) |
| --- | --- | --- | --- |
| *healthy* | 2.2 | 18 | 282 |
| *test 1* | 4.4 | 40 | 350 |
| *test 2* | 8.8 | 60 | 450 |
| *test 3* | 15 | 90 | 600 |
| *RA parameters* | 30 | 162 | 705 |
| *test 4* | 60 | 300 | 1200 |
